# Rare Presentation of Metastatic Breast Cancer Involving the Peritoneal Cavity: Two Cases Arising From Stage 0/1 Disease

**DOI:** 10.7759/cureus.86236

**Published:** 2025-06-17

**Authors:** Emily Saurborn, Jessia Adkins, Waqas Mahmud, Logan M Lawrence, Krista L Denning, Mary Legenza, Diane Krutzler-Berry

**Affiliations:** 1 Surgery, Marshall University Joan C. Edwards School of Medicine, Huntington, USA; 2 Pathology, Marshall University Joan C. Edwards School of Medicine, Huntington, USA; 3 Breast Oncology, Marshall University Joan C. Edwards School of Medicine, Huntington, USA

**Keywords:** breast cancer (bc), breast cancer metastasis, hormone receptor-positive breast cancer, peritoneal metastasis, recurrent breast cancer

## Abstract

Ductal cell carcinoma in situ (DCIS) is a noninvasive stage 0 breast cancer that arises from an abnormal proliferation of ductal epithelial cells. If untreated, it can progress to invasive ductal carcinoma (IDC), the most common form of breast cancer. A minority of women with early-stage breast cancer may experience recurrent advanced cancer, which can progress to metastatic disease, commonly in the bone, liver, lung, and brain. Improved surveillance and raised awareness over the last three decades have resulted in an increased incidence of disease; however, early detection and treatment of DCIS and IDC have a favorable prognosis. We present two cases of well-treated early-stage breast cancer with late recurrence of distal metastasis involving the peritoneal cavity and liver, with ascites as a primary presentation. In Case one, Stage 1 IDC was detected on a routine mammogram and was well-treated with lumpectomy and sentinel lymph node biopsies, chemotherapy, and radiation, with repeat mammograms negative for any evidence of recurrence. One year following treatment, the patient presented with dull epigastric pain and ascites positive for malignancy, with primary breast origin. In Case two, the patient presented to the emergency department with right upper quadrant pain and abdominal distension. A CT scan identified multiple liver lesions, and a biopsy revealed primary breast origin. A subsequent mammography detected DCIS in the right breast. One month later, the patient presented with abdominal and pelvic ascites and rapid decline of mental status before treatment was initiated. These cases underscore the importance of educating patients on self-examinations and yearly mammograms. Additionally, it is essential to educate providers on risk factors of metastatic disease and their possible presentations, including metastasis into the peritoneal cavity, to ensure optimal clinical outcomes.

## Introduction

Ductal cell carcinoma in situ (DCIS) is a stage 0 breast cancer resulting from abnormal proliferation of cells confined to the myoepithelial basement membrane of the ductal-lobular unit. According to the American Cancer Society, DCIS accounts for one in every five breast cancers [1]. Detection and diagnosis have risen since the 1980s due to increased rates of screening, and it is estimated that one in every 1300 mammograms results in a diagnosis of DCIS [2]. Although classified as a non-invasive form of breast cancer, if left untreated, 25-60% of cases of DCIS will progress to invasive ductal carcinoma (IDC) [3]. Risk factors for the recurrence of DCIS include young age (less than 40 years old) and symptomatic detection [4]. Currently, there is no way to determine which DCIS cases will progress to IDC; however, a greater risk of rare distant metastasis is correlated with poorly differentiated types of DCIS and the presence of systemic inflammation at the time of diagnosis [4, 5].

IDC is the most common type of breast cancer and accounts for 50-70% of all cases of invasive breast cancer [6]. IDC occurs when malignant cells infiltrate into the adjacent breast stroma and produce an inflammatory response in the tissues [7]. This inflammatory response results in fibrotic changes and a subsequent mass that can be palpated on a breast exam [6]. On mammography, these masses have an irregular, stellate shape. Malignant proliferation of breast epithelial cells occurs due to the overexpression of a receptor, which can include an estrogen receptor (ER), progesterone receptor (PR), or human epidermal growth factor receptor 2 (HER2). It should also be noted that the development of breast cancer is influenced by an individual’s genetic and environmental factors. In 10-15% of cases, the tumor will be triple negative (ER-/PR-/HER2-), where the tumor does not overexpress any of the receptors [8,9]. Triple negative tumors are the most aggressive subtype and have the worst prognosis [8]. After diagnosis of breast cancer, it is important to obtain an immunohistochemical stain of the tissue to determine which receptor is overexpressed so that appropriate treatment may be initiated.

Invasive breast cancer is divided into Stages 0-IV depending on the tumor, node, metastasis (TNM) staging system that analyzes the size of the primary tumor, local invasion, lymph node involvement, and metastasis to distant organs. Stage 0 tumors have malignant cells confined to the basement membrane; therefore, no cancer cells are present in the lymph nodes. Stage I tumors have invaded the basement membrane, but are still less than 2 cm, and have no lymph node involvement. In addition to receptor status, determination of grade is important in the prognosis and assessment of the patient. Grade is determined based on pathological examination of the cancer cells and considers the glandular/tubular differentiation, nuclear pleomorphism, and mitotic count. There are three distinct grades for breast cancer, with lower grades having the most favorable prognosis. Grade 1 cancers have a low mitotic rate, and the cancer cells are formed from well-differentiated glandular cells, while grade 3 or high-grade cancer cells have a high mitotic rate, poorly differentiated cells, and marked nuclear irregularities. Prognosis and treatment modalities are primarily dependent on tumor stage, grade, and characteristics of the tumor cells.

Stage 0 tumors are treated with surgery followed by radiation to eradicate the cancer cells, preventing invasive carcinoma and decreasing the rate of recurrence. Typically, adjuvant hormone therapy should be given to patients who are ER-positive. Stage I tumors are treated with a lumpectomy or mastectomy followed by radiation and possible adjuvant chemotherapy and/or hormonal therapy if the patient qualifies. Treatment modalities are dependent on patient age, comorbidities, and tumor characteristics and consist of a combination of surgery, chemotherapy, immunotherapy, hormonal therapy, and/or radiation. A histological assessment should be performed after surgical resection to ensure negative margins. If margins are positive, patients should undergo re-excision or mastectomy. After treatment of stage 0 or stage 1 breast cancer, a follow-up physical examination should be given every six to 12 months for the first five years and then annually, along with an annual diagnostic mammogram [10].

## Case presentation

We present two different cases of Stage 0/1 breast cancer with metastasis to the liver and peritoneal cavity found shortly after diagnosis or treatment. 

Case one

**Figure 1:**
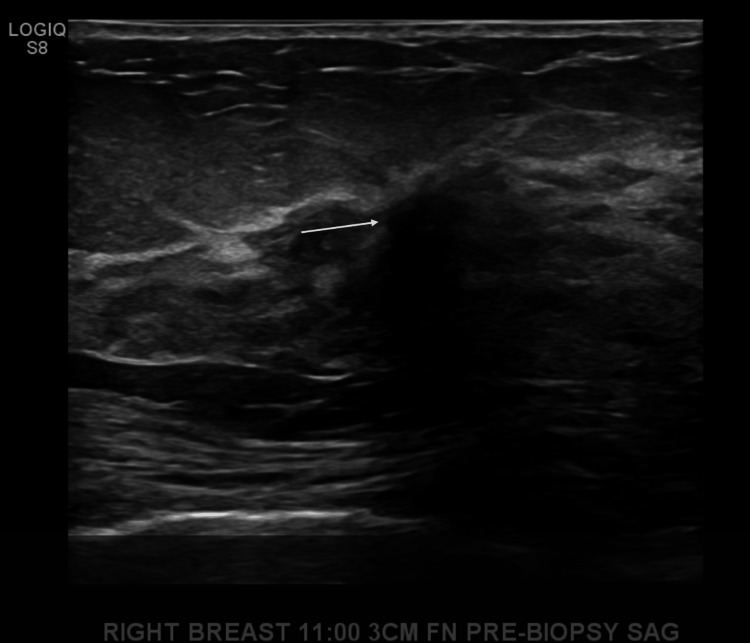
Ultrasound of the right breast from case one. Shows a hyperechoic focus with ill-defined borders measuring 6 x 8 x 6 mm in the 11 o’clock position, 3 cm from the nipple.

**Figure 2:**
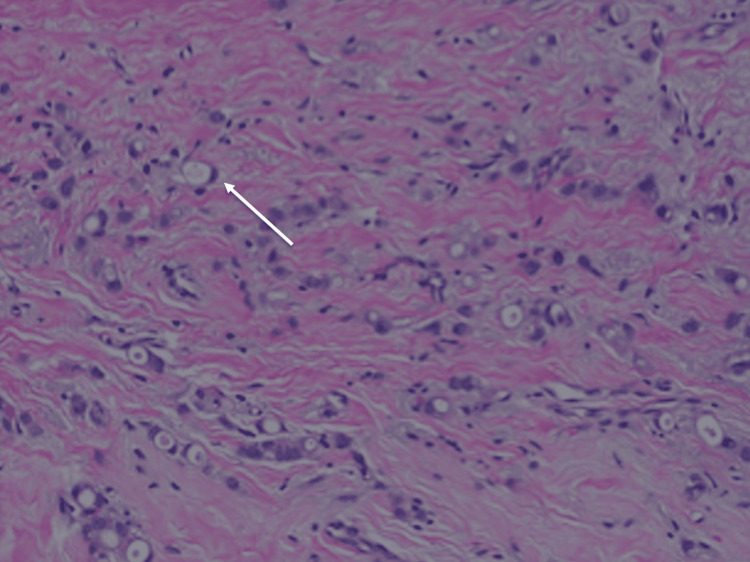
Breast biopsy. Shows invasive ductal carcinoma, with some cells displaying signet ring features. Reports revealed a low positive (4.2% weak positivity) for estrogen receptor (ER) and progesterone receptor (PR), and human epidermal growth factor receptor 2 (HER2) negative.

The first case is a 66-year-old white female who initially was well-treated for invasive ductal carcinoma. The patient first presented after a routine screening mammography in September 2021 that revealed an area of questionable architectural distortion within the upper outer quadrant of the right breast and microcalcifications within the superior aspect of the left breast. Ultrasound of the right breast demonstrated a hyperechoic focus with ill-defined borders measuring 6 x 8 x 6 mm in the 11 o’clock position, 3 cm from the nipple (Figure 1). Biopsy of the right breast revealed grade 2 IDC with low ER-positivity (4.2%), and was otherwise PR- and HER2-negative (Figure 2). Calcifications were also identified on pathology from the right breast.

**Figure 3:**
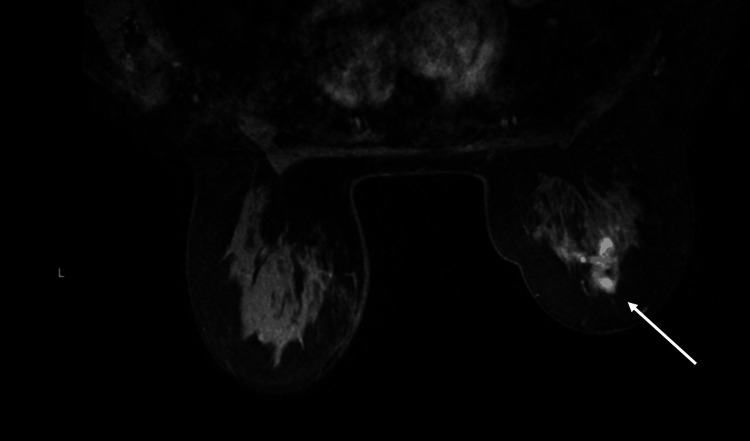
MRI from case one demonstrating a lesion in the right breast.

Bilateral MRI confirmed the mass at the 11 o'clock position corresponding to the known malignancy (Figure 3). The patient underwent a right breast lumpectomy with sentinel lymph node biopsy. Pathology of the breast tissue revealed a 15 mm invasive ductal carcinoma. The lateral margin was focally positive. Re-excision of the lateral margin and pathology results identified negative margins with no malignancy. Oncotype scoring was performed, and the results showed a recurrence score of 22, which is considered low risk, meaning that the patient would not likely benefit from chemotherapy. Her distant recurrence rate was 8%. However, the patient wanted to start chemotherapy, and after multidisciplinary discussion, it was believed that the addition of chemotherapy may be essential in this patient’s treatment process due to low ER positivity. 

The patient was started on anastrozole and was to begin radiation therapy. The patient discontinued anastrozole after two weeks due to hypertension and headaches. Subsequently, an adjuvant chemotherapy regimen was initiated, in addition to endocrine therapy, that consisted of taxotere 75 mg/m2 IV and cyclophosphamide 600 mg/m2 IV every three weeks for four cycles. After her first cycle, she developed nausea, vomiting, generalized weakness, diarrhea, low back pain, and a localized macular rash on her left wrist. She also developed neutropenia. Her dose was reduced by 15% for her second cycle, which was better tolerated. After chemotherapy, she then completed whole breast radiotherapy beginning in April 2022, after which she began taking letrozole. Due to severe scalp itchiness, letrozole was switched to exemestane. A bilateral MRI of the breast performed in January 2023 was negative for malignancy or lymphadenopathy. A screening mammography in November 2023 revealed extremely dense breast tissue, benign calcification in the left breast, and no evidence of malignancy. 

**Figure 4:**
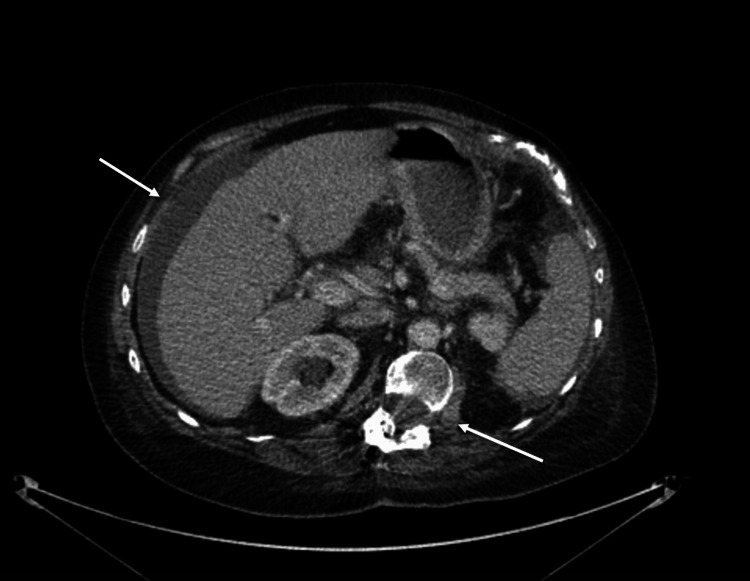
CT abdomen and pelvis from case one demonstrating abdominal ascites with retroperitoneal inflammation.

In January 2024, the patient presented to the emergency department for acute abdominal pain and generalized malaise. Labs revealed elevated liver transaminases, leukopenia, thrombocytopenia, and neutropenia. CT scan of the abdomen and pelvis demonstrated abdominal ascites around the liver and the spleen extending along the paracolic gutters to the pelvis, prominence of the renal collecting system bilaterally, and significant inflammation at the retroperitoneum on the left side (Figure 4). An abdominal ultrasound showed cholelithiasis and gallbladder wall thickening, which is likely chronic, fatty infiltration of the liver, and right upper quadrant ascites. CT-guided paracentesis reported elevated white blood cells and malignant cells. Tumor markers, cancer antigen (CA) and carcinoembryonic antigen (CEA), were found to be elevated. For treatment, she was started on Xeloda. Repeat CT scan of the abdomen and pelvis revealed a slight increase in ascitic volume and mild wall thickening of the ascending and transverse colon, possibly indicating colitis.

MRI of the abdomen and of the pelvis in February was significant for mild ascites with no obvious evidence of metastatic disease, left hydronephrosis, thickening of the urinary bladder, and a pleural effusion in the right lung. Positron emission tomography (PET)/CT skull to the thigh showed a large amount of ascites in the abdomen with no evidence of malignant foci. Due to worsening renal function, the patient was instructed to stop Xeloda. Colonoscopy with biopsy of the ascending colon performed at this time was negative for malignancy. Serial paracenteses were performed every two weeks due to the rapid recurrence of ascites. 

**Figure 5:**
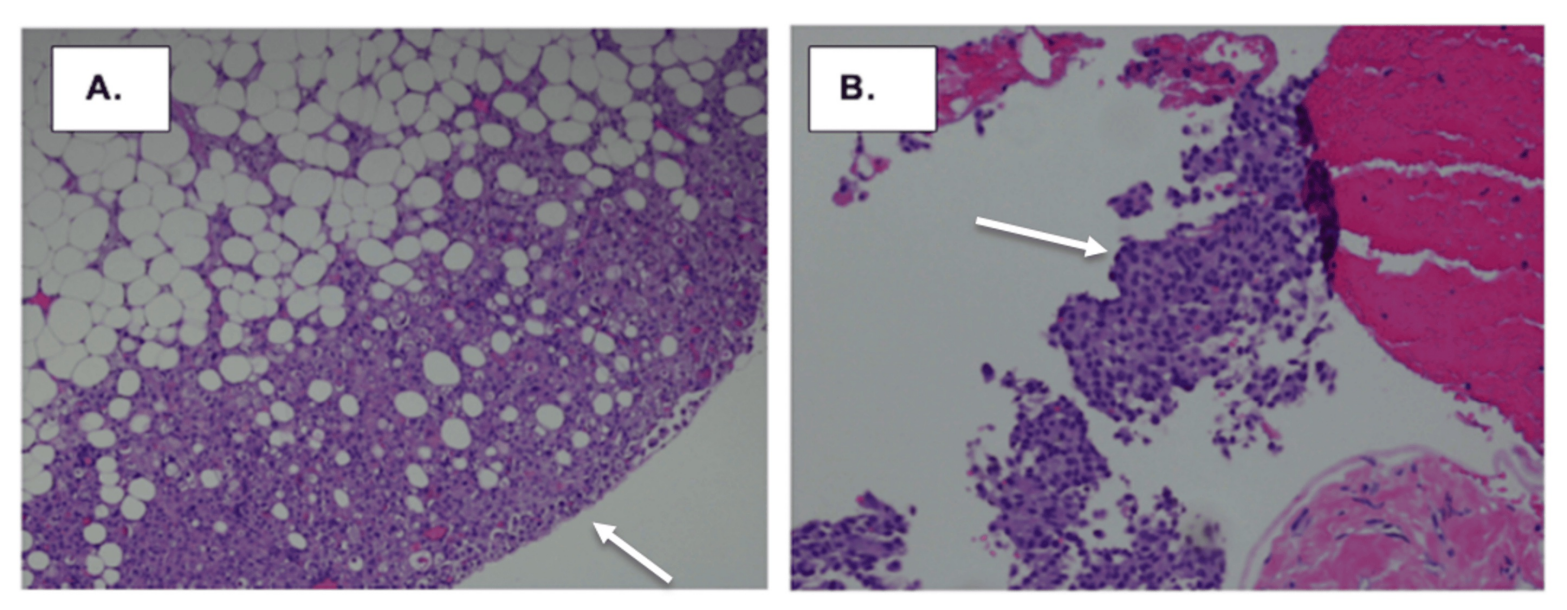
(A) Omentum and (B) peritoneum. Shows poorly differentiated malignant cells with solid and microcystic architecture. Immunohistochemical stains were performed, which supported a primary breast origin.

In March, biopsies of the sigmoid nodule and bladder peritoneum, as well as a partial omentectomy, were performed via diagnostic laparoscopy. Biopsy results revealed metastatic poorly differentiated carcinoma in the omentum and bladder peritoneum, favoring primary breast origin (Figure 5); however, the sigmoid nodule was negative for malignancy. Other studies reported that the PD-L1 tumor expression was 0%, the tumor mutation burden was 16 mutations per megabase, and the microsatellite status was stable. Genomic mutations were found in AKT1 E17K, CBFB D128fs*1, MAP2K4 T78fs*10, and TP53 R273H. There were no reportable alterations in BRCA1, BRCA2, ERBB2, ESR1, or PIK3CA. The patient started chemotherapy with paclitaxel 80mg/m2 intravenously weekly. Zoledronic Acid and pembrolizumab were added to the regimen. The patient was readmitted in July for bacteremia. During the hospital stay, the patient reported new-onset dysphagia, prompting biopsies of the esophagus and stomach. Pathology results confirmed the diagnosis of metastatic carcinoma, and the patient opted to enroll in hospice. 

Case two

**Figure 6:**
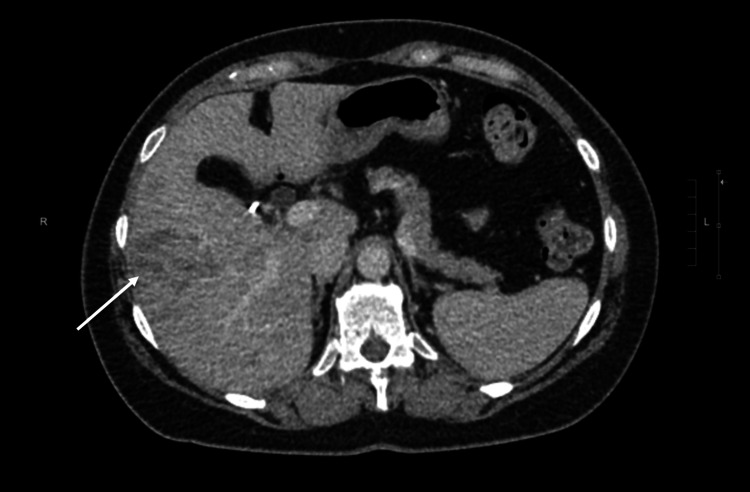
CT abdomen from case two showing metastasis in the right lobe of the liver.

The second case involves a 49-year-old female who presented to the emergency department in March 2024 with right upper quadrant pain that she thought was appendicitis. The patient endorsed pain with deep breathing, constipation, and abdominal distension that had been present for one to two months. A CT scan ordered in the emergency department revealed numerous ill-defined, patchy, and nodular areas of hypodensity throughout the right lobe of the liver (Figure 6). The most dominant rounded area measured approximately 7.0 x 5.8 x 6.3 cm. Further, an enlarged lymph node was visualized on CT. CT-guided biopsy of the liver mass was significant for a GATA-3 positive, CK7 positive, ER negative, PR negative, and HER2 positive lesion, suggestive of a metastatic process with a primary lesion originating in the breast. Her oncologist felt that she was a candidate to undergo palliative chemotherapy with the following regimen of trastuzumab plus pertuzumab and taxane, based on the Cleopatra trial.

**Figure 7:**
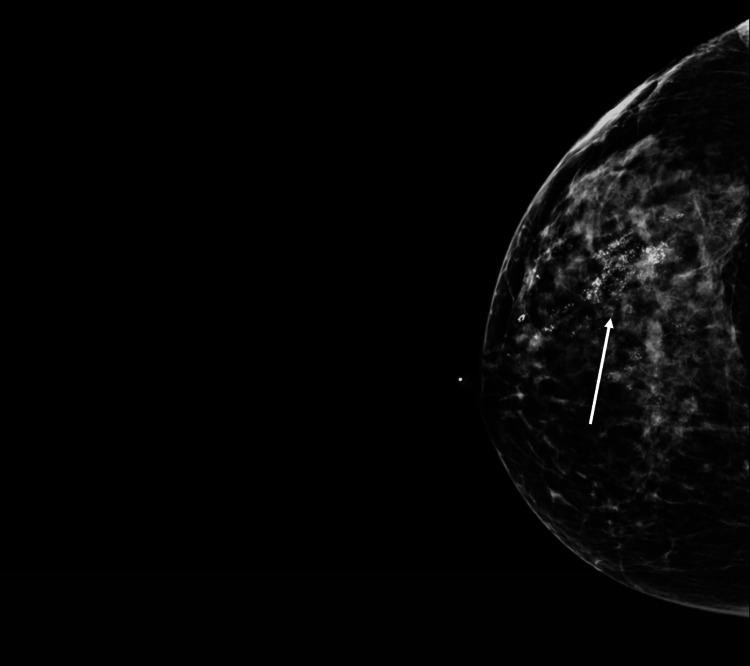
Craniocaudal (CC) mammogram of the right breast from case two demonstrating extensive calcifications.

**Figure 8:**
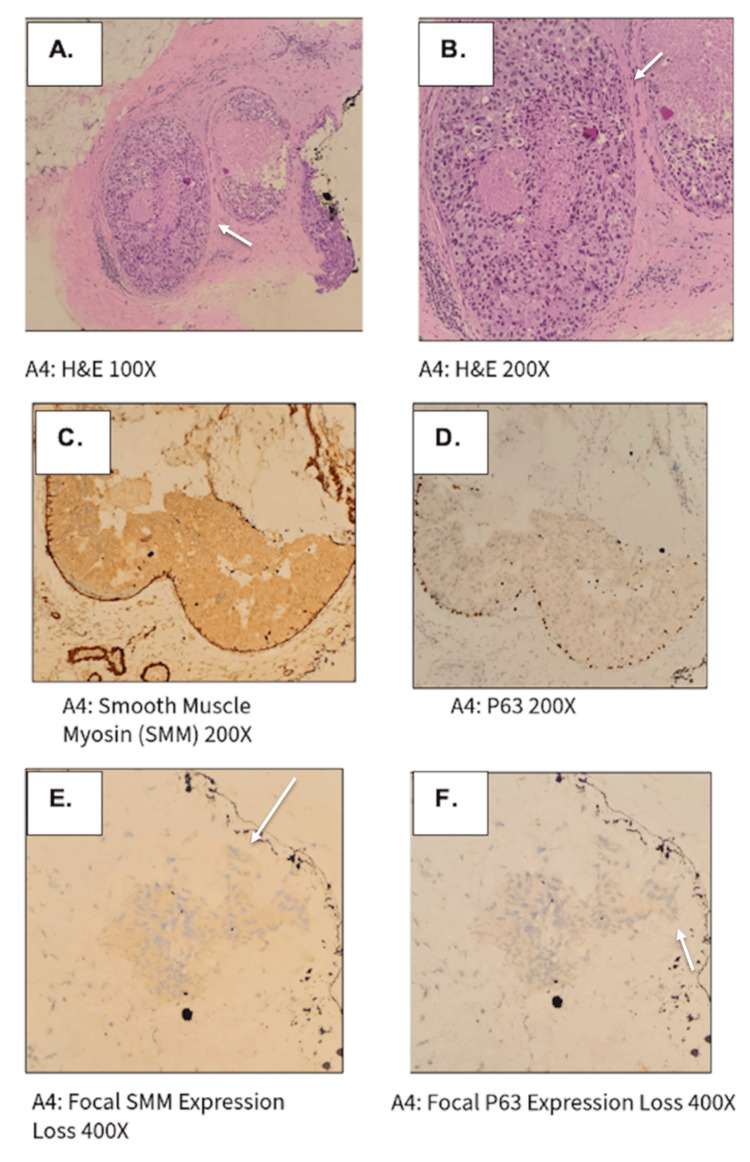
Pathology results from stereotactic biopsy. Revealed (A, B) ductal carcinoma in situ (DCIS), high-grade, solid type, with calcification and necrosis measuring at least 3 mm, with a focal area suspicious for microinvasive carcinoma. (C) By immunohistochemistry, smooth muscle myosin and (D) p63 highlight myoepithelial cells around DICS. (E) Focal area suspicious for microinvasion showing loss of expression for smooth muscle myosin and (F) p63.

A workup to identify a primary breast lesion was performed in April 2024. Bilateral diagnostic mammogram revealed extensive calcifications in the lower outer right breast and a question of architectural distortion in the upper outer left breast (Figure 7). A 1.2 cm heterogeneous mass in the left upper outer breast in the 3 o’clock position, 7 cm from the nipple, was also reported. Physical exam findings of the right breast had a 6 cm area of thickening in the lower outer region with skin retraction and lateralization of the nipple. The patient underwent a biopsy of the lower outer calcifications in the right breast. Pathology reports revealed high-grade DCIS of the solid type with calcifications and necrosis (Figure 8). There was also a focal area that was highly suspicious for microinvasive carcinoma.

In May, the patient presented to the emergency department for worsening abdominal pain, swelling, constipation, decreased appetite, shortness of breath, and nausea. A CT ordered by the emergency department revealed abdominal and pelvic ascites, hepatomegaly, hepatic metastatic disease, and a small nodule present in the inferior right breast measuring 1 cm. Diagnostic paracentesis performed reported inflammatory cells and debris, but was negative for any malignant infiltration. A CT scan of the head and brain was ordered due to altered mental status, and the results were unremarkable.

**Figure 9:**
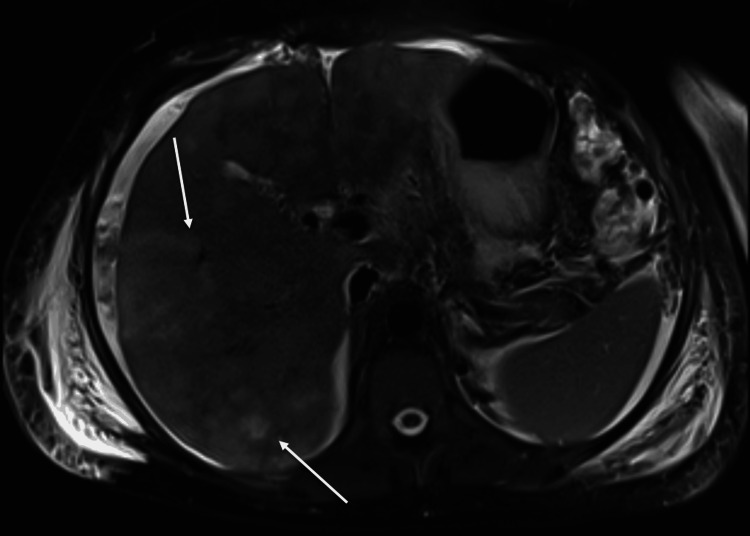
MRI abdomen from case two demonstrating diffuse metastasis to the liver and ascites.

**Figure 10:**
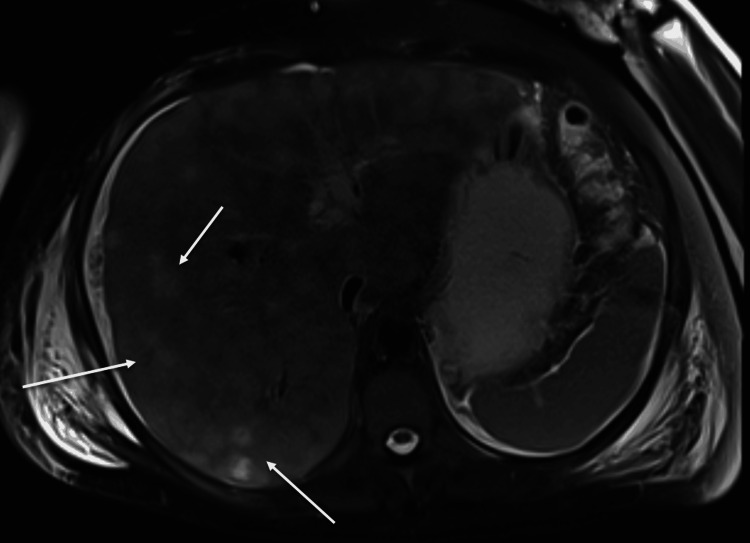
MRI abdomen from case two showing diffuse hepatic metastatic disease and ascites.

The night following the CT scan, the patient complained of having hallucinations. An MRI of the brain was performed and did not show any evidence of metastatic disease. MRI of the abdomen and magnetic resonance cholangiopancreatography (MRCP) of the pelvis were performed for jaundice and revealed diffuse hepatic metastatic disease, ascites, and portacaval adenopathy (Figures 9, 10). The next day, the patient lost her ability to communicate and developed acute metabolic encephalopathy due to hyperammonia. An nuclear medicine (NM) whole body scan ordered at this time was negative for any metastatic uptake within bone. Unfortunately, due to the severity of the patient's condition and her rapid decline, it was agreed that the patient would not receive any more chemotherapy and that home hospice comfort care would be in her best interest.

## Discussion

Breast cancer rates continue to increase annually by 0.6%; however, mortality rates continue to decrease due to mammographic detection and raised awareness. It is estimated that in 2024, there will be 310,720 females diagnosed with breast cancer and 42,250 deaths as a result [11]. Due to increased survival rates, physicians need to detect distant recurrence, which is a significant contributor to disease-related mortality. It is estimated that 30% of women who were initially diagnosed with early-stage breast cancer are projected to experience recurrent advanced cancer, ultimately progressing to metastatic disease with time [12]. Breast carcinoma metastasis is likely to occur in the bone, liver, lung, and brain, with other sites being less common. Metastasis to the peritoneum is rarer, and when this does occur, it is to a significantly greater extent from invasive lobular carcinoma (ILC) than IDC [13].

Peritoneal metastasis with ascites as primary presentation is a rare complication of metastatic breast cancer, yet few cases have been reported in the literature. Patwari et al. reported a case of a 65-year-old woman who presented to the emergency department with symptoms of nausea and abdominal pain [14]. On physical exam, the patient had shifting dullness, and CT revealed large volume ascites. The patient had no previous history of breast cancer. Bone marrow biopsy was significant for malignant cells positive for pancytokeratin AE1/AE3, cam 5.2, GATA-3, estrogen receptor, and HER2 negative. AE1/AE3 positive cells and cam 5.2 positive cells are consistent for epithelial-derived tumors, while GATA-3 positive cells indicate a primary breast origin [15]. The patient’s mammogram, however, was negative for any evidence of malignant masses or microcalcifications. Interestingly, Mostafa et al. reported a case of a 54-year-old woman who presented with gastrointestinal symptoms and ascites [16]. The patient’s past medical history included liver cirrhosis due to nonalcoholic fatty liver disease. Additionally, the patient’s past medical history was significant for stage IIA (cT2cN0M0) high-grade invasive ductal carcinoma, positive for ER and PR receptors. The patient underwent neoadjuvant chemotherapy followed by lumpectomy with negative sentinel lymph nodes. Radiotherapy followed by a five-year course of hormonal therapy was completed one year prior to disease recurrence. In 2021, the patient had recurrent DCIS of the left breast and underwent a nipple sparing mastectomy. At the time of mastectomy, the lymph nodes were negative for metastatic processes. In the same year, the patient underwent a hysterectomy and bilateral salpingo-oophorectomy for adenomyosis, and no peritoneal involvement was seen at that time. In 2023, the patient was admitted for upper gastrointestinal (GI) hemorrhage and significant ascites. Paracentesis of the fluid revealed malignant cells of breast origin. The patient was treated with Palbociclib and Letrozole. Repeat CT after three months of treatment demonstrated stable omentum illness and only minor ascites. Due to the regression of the disease, Palbociclib was discontinued. The patient continued to have stable imaging with treatment of Letrozole alone. 

Further, Al Qahtani et al. reported a case of a 26-year-old female who presented to the emergency Department with cholestatic jaundice, coagulopathy, and ascites [17]. The patient reported a three-month history of moderate-to-severe, dull aching epigastric pain. The patient was admitted, and a workup to rule out autoimmune, infectious, or malignant etiologies was performed. Abdominal ultrasound revealed liver cirrhosis, with an irregular-shaped lesion infiltrating the right lobe. CT performed reported multiple hypodense masses and ascites. Paracentesis reported a large number of white blood cells, and the diagnosis of spontaneous bacterial peritonitis was made. Biopsy of the lesion revealed adenocarcinoma, likely secondary to breast origin. The patient reported feeling a breast mass a few years prior but never sought treatment at the time. The patient passed away on hospital day 28 secondary to liver failure with lactic acidosis and DIC. Due to the patient’s severe metastatic involvement of the liver and liver failure, chemotherapy was never initiated. Mostafa et al. reported a case of a patient who began chemotherapy shortly after peritoneal involvement and was able to achieve disease regression, while Al Qahtani et al. reported a case of a patient who presented too late in the disease course. Metastasis to the peritoneal cavity has a poor prognosis, and early detection is critical in the treatment course for patients [18]. Currently, treatment options for peritoneal metastasis are lacking, but as more cases occur, it is hoped that a successful regimen may be implemented. 

Although involvement of the peritoneal cavity is a rare complication of metastatic breast cancer, there is ample evidence to suggest that it does occur. Breast cancer recurrence rates are difficult to predict; however, it is important to counsel patients on the importance of yearly screening exams. Risk factors for breast cancer include obesity, nulliparity, early age of menses, late menopause, family history, ethnicity, and age, the most important risk factor. Risk factors for recurrent breast cancer include the presence of biological markers of Ki-67 and TP53, lifestyle factors, BRCA1 or BRCA2 mutations, younger age, obesity, lymph node involvement, and histologic grade of the tumor cells [14,19]. Further, patients with hormone-positive tumors may have a higher risk of later recurrence if the primary tumor had a poor response to treatment, and if the patient failed to receive their recommended radiation therapy [20]. It is important to educate patients on their independent risk factors of breast cancer and encourage self-breast examinations and annual imaging. Metastasis to the peritoneal cavity has a poor prognosis, and early detection is critical in the treatment course for patients. Therefore, it is imperative to counsel patients on yearly screenings and yearly follow-ups to prompt any early workups if necessary. 

## Conclusions

Although ascites secondary to peritoneal metastasis as a primary presentation for metastatic breast cancer is rare, there is evidence to suggest its occurrence. While early diagnosis of primary breast cancer with peritoneal metastasis may not improve survival rates, it will allow for earlier initiation of treatment, ultimately improving quality of life for patients. Further, this case underscores the importance of routine preventative screening and consistent follow-up with a multidisciplinary team following a breast cancer diagnosis.
